# A Bio-inspired Collision Avoidance Model Based on Spatial Information Derived from Motion Detectors Leads to Common Routes

**DOI:** 10.1371/journal.pcbi.1004339

**Published:** 2015-11-19

**Authors:** Olivier J. N. Bertrand, Jens P. Lindemann, Martin Egelhaaf

**Affiliations:** Neurobiologie & CITEC, Bielefeld University, Bielefeld, Germany; Technische Universitat Chemnitz, GERMANY

## Abstract

Avoiding collisions is one of the most basic needs of any mobile agent, both biological and technical, when searching around or aiming toward a goal. We propose a model of collision avoidance inspired by behavioral experiments on insects and by properties of optic flow on a spherical eye experienced during translation, and test the interaction of this model with goal-driven behavior. Insects, such as flies and bees, actively separate the rotational and translational optic flow components via behavior, i.e. by employing a saccadic strategy of flight and gaze control. Optic flow experienced during translation, i.e. during intersaccadic phases, contains information on the depth-structure of the environment, but this information is entangled with that on self-motion. Here, we propose a simple model to extract the depth structure from translational optic flow by using local properties of a spherical eye. On this basis, a motion direction of the agent is computed that ensures collision avoidance. Flying insects are thought to measure optic flow by correlation-type elementary motion detectors. Their responses depend, in addition to velocity, on the texture and contrast of objects and, thus, do not measure the velocity of objects veridically. Therefore, we initially used geometrically determined optic flow as input to a collision avoidance algorithm to show that depth information inferred from optic flow is sufficient to account for collision avoidance under closed-loop conditions. Then, the collision avoidance algorithm was tested with bio-inspired correlation-type elementary motion detectors in its input. Even then, the algorithm led successfully to collision avoidance and, in addition, replicated the characteristics of collision avoidance behavior of insects. Finally, the collision avoidance algorithm was combined with a goal direction and tested in cluttered environments. The simulated agent then showed goal-directed behavior reminiscent of components of the navigation behavior of insects.

## Author Summary

The number of robots in our surroundings is increasing continually. They are used to rescue humans, inspect hazardous terrain or clean our homes. Over the past few decades, they have become more autonomous, safer and cheaper to build. Every autonomous robot needs to navigate in sometimes complex environments without colliding with obstacles along its route. Nowadays, they mostly use active sensors, which induce relatively high energetic costs, to solve this task. Flying insects, however, are able to solve this task by mainly relying on vision. Any agent, both biological and technical, experiences an apparent motion of the environment on the retina, when moving through the environment. The apparent motion contains entangled information of self-motion and of the distance of the agent to objects in the environment. The later is essential for collision avoidance. Extracting the relative distance to objects from geometrical apparent motion is a relatively simple task. However, trying to accomplish this with biological movement detectors, i.e. movement detectors found in the animal kingdom, is tricky, because they do not provide unambiguous velocity information, but are much affected also by the textural properties of the environment. Inspired by the abilities of insects, we developed a parsimonious algorithm to avoid collisions in challenging environments solely based on elementary motion detectors. We coupled our algorithm to a goal direction and then tested it in cluttered environments. The trajectories resulting from this algorithm show interesting goal-directed behavior, such as the formation of a small number of routes, also observed in navigating insects.

## Introduction

Anyone who has tried to catch flying flies will be familiar with their amazing performance. Within a fraction of a second, flies perform high-speed turns to avoid a predator or a collision with an obstacle. The collision avoidance decisions are produced in a fly’s brain with very limited neural resources [1, 2] and are transformed into an evasive turn within only a few milliseconds, a rather short time compared to human reaction times [3]. As such, flying insects have become an important model system for understanding the minimal computation requirements for spatial vision tasks, such as collision avoidance [4]. Engineers are also looking for fast and cheap collision avoidance algorithms, without the use of expensive devices, e.g. 3D laser rangefinders [5], or extensive computations, e.g. Lucas-Kanade optic-flow computation [6]. Any motion of an agent, such as an insect or a robot, induces apparent movement of the retinal image of the surroundings, i.e. optic flow. The optic flow experienced during translations in a static environment depends on the agent’s speed, its nearness to objects and its motion direction. When the agent moves fast or close to objects, the optic flow amplitude will be high. By contrast, the rotational optic flow depends only on the ego-motion of the agent and, thus, is independent of the spatial layout of the environment. Information on the nearness of objects is relevant for determining a collision avoidance direction. Therefore, the translational optic flow can be exploited for collision avoidance. Flies, and also other insects and some birds, show an active gaze strategy, which separates the self-motion into saccades (i.e. mainly rotation) and intersaccades (i.e. mainly translation) [7–15]. The saccade amplitude of an insect or a bird is thought to be driven, at least in the vicinity of potential obstacles, by the optic flow gathered during the translation preceding the saccade.

Insects estimate the optic flow with correlation-type elementary motion detectors (EMDs), a concept first introduced by Reichardt and Hassenstein in the 1950s [16]. A characteristic property of the EMD is that its output does not exclusively depend on velocity, but also on the pattern properties of the stimulus, such as its contrast and spatial frequency content. Therefore, the nearness, extracted from optic flow estimated by insects, is expected to be entangled with properties of the textures of the environment. Visual-oriented tasks based on optic flow, such as collision avoidance, might, therefore, be a challenge. Several mechanisms of collision avoidance have been proposed based on behavioral experiments on various insect species [14, 17–23]. However, these models have not yet been shown to be functional under a wide range of conditions, or do not use optic flow measured by correlation-type elementary motion detectors.

In the present paper, we propose a model of collision avoidance based on EMDs which will be shown to be successful in various environments. The model of collision avoidance can be subdivided into three processing steps: (1) extraction of nearness from optic flow, (2) determination of a collision avoidance direction from the map of nearness estimations, i.e. where to go, and (3) determining a collision avoidance necessity, i.e. whether it is dangerous not to follow the collision avoidance direction. The nearness measurements will be shown to be proportional to a pseudo-norm of the optic flow, independent of the direction of motion, as long as the agent moves in a plane and has a spherical eye. The collision avoidance direction and necessity will be computed via spatial integration of the nearness. The collision avoidance algorithm will, firstly, be tested with geometrical optic flow, i.e. a measure of optic flow independent of object texture, to build a benchmark and show that optic flow information is sufficient to solve the problem. Then, EMDs will be used and the algorithm will be challenged in different environments. Finally, we will show that the collision avoidance algorithm based on EMDs can be coupled with a navigation direction in order to reach a given location without colliding with obstacles along the trajectory.

## Results

### Optic flow and relative nearness

When a distant object is approached at a high speed, the situation might be as dangerous as when a close object is approached at a slower speed. The relative nearness, i.e. the nearness times the agent’s speed, can be seen as a measure of how soon the agent will collide with the object when the agent moves in the direction towards where the measurement was performed. This information is highly relevant for collision avoidance. Since the relative nearness is linked to the optic flow, the first step of the collision avoidance algorithm is to transform the optic flow into relative nearness. The translational optic flow, i.e. the optic flow experienced during the brief translatory phases of self-motion modeled after the intersaccadic intervals of insect flight, is determined jointly by the agent’s self-motion and the three-dimensional structure of the environment. The independent extraction of these two parameters entangled in the optic flow field is challenging [24]. We will show that the three-dimensional structure of the environment can be extracted from translational optic flow if the translation is confined to a plane and the eye of the agent is spherical.

The optic flow field is a two-dimensional vector field, where each vector is the apparent velocity of the objects on the eyes of the agent. The optic-flow field experienced during translation results from the product of the relative nearness of objects in the environment and a factor depending on the angle between the direction of self-motion and the direction in which these objects are seen (“viewing angle”). A transformation removing the factor depending on the viewing angle is required to extract relative nearness from optic flow. The dependence of this factor on the viewing angle can be understood best when the relative nearness is constant for the entire visual field, i.e. when the agent is placed in the center of a sphere and moves in the equatorial plane. The optic-flow field for a spherical eye can be expressed for each point in the visual field in terms of the vertical flow component, i.e. the flow along the elevation, and the horizontal flow component, i.e. the flow along the azimuth. The horizontal flow component, experienced during a translation in the equatorial plane in the center of a sphere, increases from the front to the side (i.e. 90° away from the motion direction) and then decreases again towards the back (S1B Fig). The horizontal flow is independent of the elevation (see S1 Text,Eq.S5). Respectively, the vertical optic-flow component decreases from the front to the side, and then, increases again towards the back. By contrast, the vertical flow is not symmetric by rotation around the direction of motion and, therefore, depends on the elevation (see S1 Text,Eq.S5). It increases from the equator to the poles (S1A Fig). Therefore, the horizontal flow and the vertical flow have an antagonistic variation from the front to the back. Due to the assumption that the movement of the agent is constrained to the equatorial plane, the variation of the vertical flow with elevation does not depend on the direction of motion. This variation can, therefore, be corrected (see S1 Text, and S1C Fig). Interestingly, the sum of the horizontal flow squared and the corrected vertical flow squared can be shown to be independent of the viewing angle (see S1 Text, Eq. S8). The transformation will be called a retinotopically modified norm of the optic-flow field. When the agent does not move within a sphere, the result of this transformation will not be constant for every viewing angle, but equal to the product of speed (*v*) and nearness (*μ*), i.e. the relative nearness. The optic flow has two singular points, the focus of expansion (FOE) and the focus of contraction (FOC). At these two points, the result of the retinotopically modified norm of the optic flow will be null, independent of the nearness of objects.

**Fig 1:**
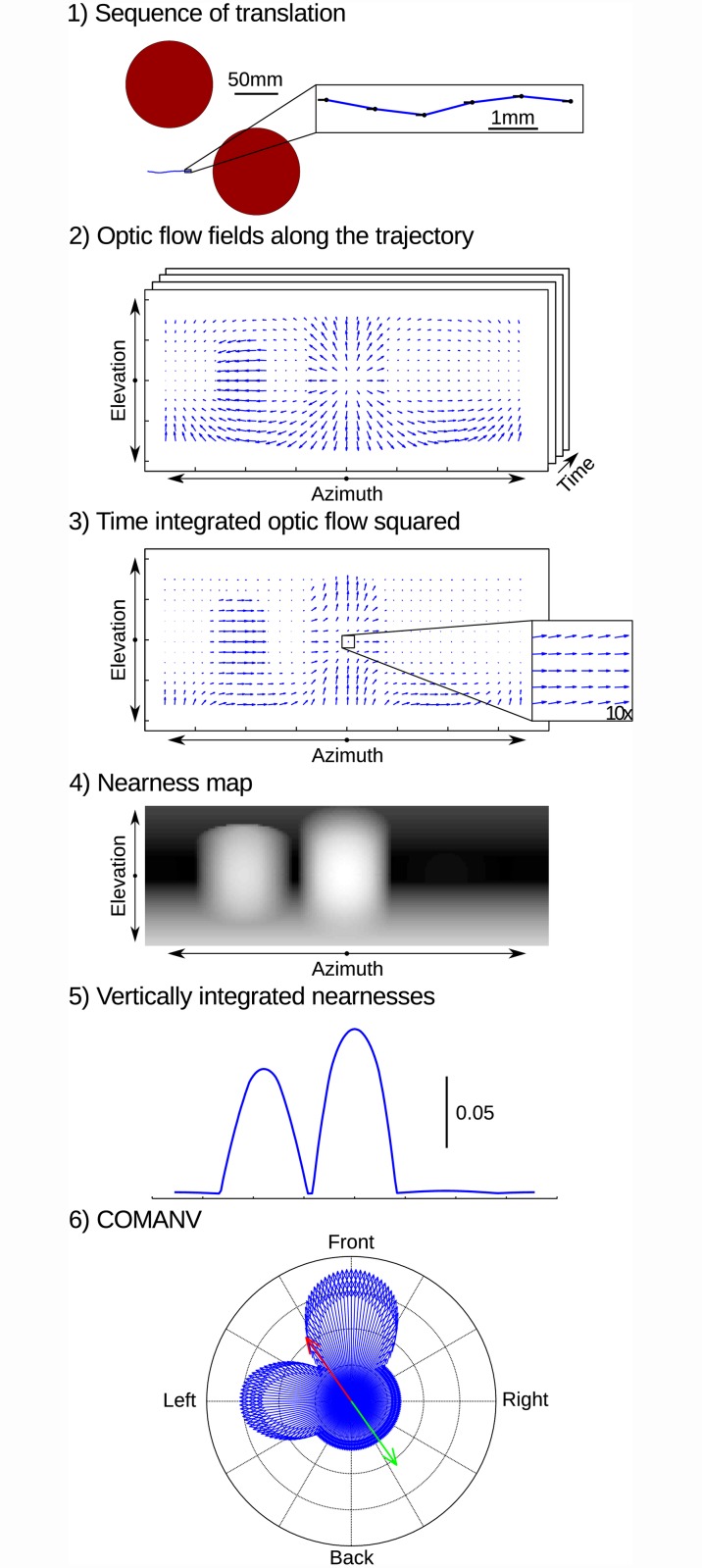
Sketch of the algorithm from motion to CAD. 1) The motion of the agent consists of a series of translations in the null elevation plane. 2) Optic-flow fields along the trajectory contain FOEs and FOCs. 3) The time-integrated optic flow squared does not contain FOC and FOE. Inset is a 10× zoom at the mean motion direction of the agent. 4) Nearness map computed from time-integrated optic flow squared. 5) Nearness map averaged along the elevation. 6) Computation of the COMANV. Blue: representation of the vertically integrated nearness map in polar coordinates. Red: vectorial sum of the vertically integrated nearness vectors (COMANV). Green: vector directed opposite to the COMANV.

**Fig 2:**
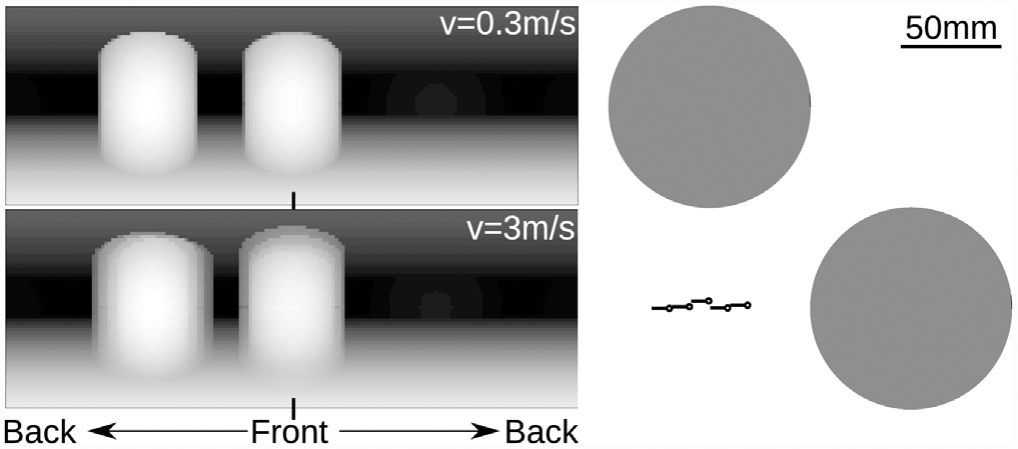
Blurred relative nearness of two cylindrical obstacles at high speed of the agent. Left panels: Nearness maps computed from optic flow experienced during translation at a speed of 0.3ms^−1^ and 3ms^−1^. Right panel: Trajectory at the speed of 3ms^−1^ towards one obstacle. Black circle and black line represent the head and the body of the agent, respectively. Gray circles represent the objects seen from above.

The relative nearness can be extracted from the optic flow independent of the viewing angle, except for the FOE and the FOC. This problem can be solved by combining translational flow-fields arising from different directions of translational movement and, thus, with different FOCs and FOEs. However, an agent cannot easily obtain several translational flow fields centered at a given point in the world. On the other hand, the nearness to objects does not strongly differ in realistic environments for two sufficiently close points in space. Therefore, let us consider an agent performing a translation composed of sub-translations in different motion directions, i.e. a combination of different forward and sideways motion components (Fig 1 part 1). Each sub-translation leads to an optic-flow field which has the retinotopically modified norm properties. The average optic-flow component obtained from the series of sub-translations also has the retinotopically modified norm properties, but does not have singular points (Fig 1 part 3). The agent can then compute the relative nearness to objects within its entire visual field by using the retinotopically modified norm of the averaged squared optic flow (Fig 1 part 4). When the object nearness for a viewing direction changes during the translation, the relative nearness map will be blurred. The longer the spatial lengths of the sub-translations are, the more the relative nearness map is blurred. This effect does not necessarily cause problems for collision avoidance, because the blurred relative nearness map still represents the overall depth-structure of the environment, though on a slightly coarser scale (Fig 2). Fig 2 shows the nearness map computed from the geometrical optic flow in an environment containing two objects. At a higher speed, the nearness map is blurred due to the integration of the geometrical optic flow over time.

### Collision avoidance with geometrical optic flow

**Fig 3:**
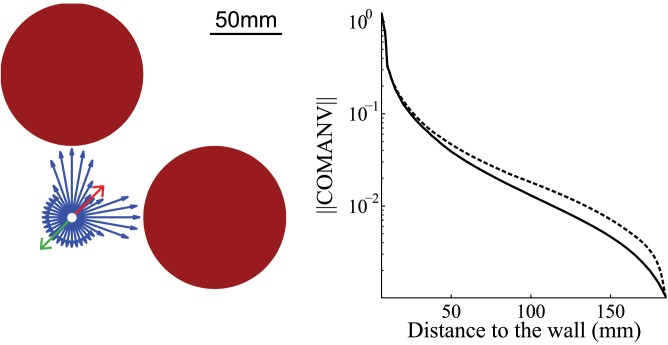
Direction and norm of COMANV. Left panel: Direction of the COMANV. Blue, red and green vectors are nearness vector, +COMANV, and -COMANV, respectively. Red disks represent the objects. (The norms of the vectors have been scaled.) Right panel: Norm of COMANV as a function of the distance to the box wall. Box height: 390mm (solid line) and 3900mm (dashed line).

Once the relative nearness map is known, in which direction the agent should move to avoid a collision (collision avoidance direction, CAD) and how important it will be to follow this direction (collision avoidance necessity, CAN) need to be determined. Based on this information, the amplitude of the necessary saccade-like turn was determined. In order to establish a benchmark for the performance of this algorithm, it was developed, firstly, on the basis of the geometrical optic flow. Only the shape of the environment along the azimuth is required to perform collision avoidance for movements in a plane. Therefore, averaging the relative nearness map along the elevation does not lead to a loss in spatial resolution along the azimuth (Fig 1 part 5) and, thus, should not affect the collision avoidance performance. As will be shown below, this averaging was especially relevant when the relative nearness map was estimated on the basis of EMD responses (see CAD and CAN from EMD). The averaged relative nearness can be represented by vectors in polar coordinates with the argument of the vectors being the azimuth and their length the relative nearness averaged along the elevation (Fig 1 part 6). The vector sum of all averaged relative nearness vectors will be termed the Center-Of-Mass Average Nearness Vector (COMANV). It points towards the average direction of close objects in the environment (Fig 3). It may, therefore, be a plausible strategy to turn in the opposite direction to the COMANV, i.e. the CAD, to avoid obstacles. This zero-order approach is, to some extent, similar to the collision avoidance algorithm used by 3D range finder robots [25]. It may lead to suboptimal trajectories. A more optimal strategy would be to pick a direction without obstructions [26]. However, this strategy would require a reliable relative nearness map provided by local self-localization and mapping [27].

**Fig 4:**
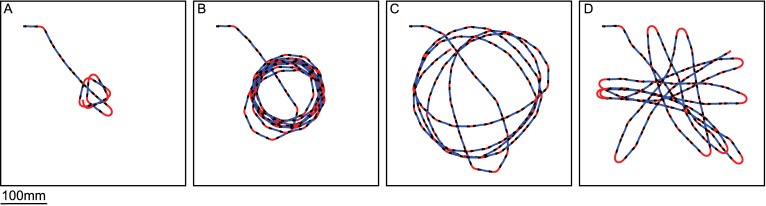
Closed-loop simulations of trajectories of the agent equipped with the collision avoidance algorithm in a cubic box. Blue and red lines are intersaccades and saccades, respectively. A) The saccade amplitudes were computed such that the agent moves in the CAD after the saccade. B, C and D) The saccade amplitudes were computed such that the agent moves in a direction corresponding to only a fraction of the CAD after the saccade. The fraction of the CAD was computed with a sigmoid function, parameterized by a gain and a threshold, of the CAN. B) Gain = 2, Threshold = 1.6. C) Gain = 2, Threshold = 3.2 D) Gain = 10^6^, Threshold = 3.2.

The argument of the COMANV provides the agent with a direction to avoid a collision by pushing it away from obstacles. Fig 4A shows a closed-loop simulation of collision avoidance in a box. The agent trajectories converge at the center of the box. Indeed, if an agent is pushed away from obstacles at every location in an environment, it ends at a point in the environment equilibrating object distances. However, collision avoidance behavior is not necessary if the obstacles are sufficiently far from the agent. To allow the agent to assess when it has to avoid an object, a measurement of CAN is required. The argument of the COMANV, the CAD, has been used, so far, to compute the saccade amplitude and, thus, to determine the agent’s new direction of motion. However, the norm of the COMANV also has interesting properties: It has the same unit as the relative nearness, i.e. the inverse of a time. Hence, the norm of the COMANV can be regarded as a measurement of the CAN: the larger the norm of COMANV, the larger is the necessity for the agent to make an evasive behavioral response. To assess the relationship between the distance to objects and the norm of the COMANV, the relative nearness map has been extracted at different distances to the wall. Since the apparent size of the objects might also affect the norm of the COMANV, different wall heights were used (Fig 3). The norm of COMANV increases with both the apparent size of an object and the nearness to it. The apparent size of the object has a smaller effect on the norm than the nearness. Thus, the norm can be used as a measurement of CAN.

Depending on the amplitude of the CAN, the agent may have one of two behavioral options. It should turn via a saccade toward the CAD calculated if the CAN is sufficiently high. Alternatively, it should continue moving straight if the CAN is smaller than a critical value. However, the CAN does not necessarily affect the behavior of the agent in an all-or-nothing fashion, i.e. making a turn according to CAD or making no turn at all. Rather, a kind of compromise may also be possible. Since the CAN is a continuous variable, the agent may turn, via a saccade, towards a direction which is a compromise between CAD and the previous direction of motion. The compromise can be modeled as a weight given by a sigmoid function of the CAN (see Materials and Methods). The saccade amplitude is then the product of this weight and CAD. The sigmoid function of CAN is parameterized by a threshold and a gain. The gain controls how much the saccade amplitude corresponds to CAD. A high gain will approximate a behavior with two distinct states: “turn, by a saccade, toward CAD” or “continue moving straight” (Fig 4D). A small gain will, however, generate a smooth transition between the two behaviors, modeling a decreasing saccade amplitude with decreasing CAN (Fig 4C). The threshold determines the border between the zone in the environment where saccade amplitudes are mainly driven by CAD, i.e. collision avoidance is necessary, and the zone where the saccade amplitudes are mainly driven by the previous direction of motion, i.e. collision avoidance is not necessary (S3 Fig). The effect of the threshold can be seen by comparison Fig 4B and 4C.

In summary, the collision avoidance algorithm uses the COMANV to determine the collision avoidance direction CAD and to change the behavior of the agent. The algorithm can be subdivided into five steps.

1. Extract relative nearness map from optic flow during an intersaccade composed of a mixture of different forward and sideways motion components.
2. Compute the COMANV from the relative nearness map.
3. Extract the CAD (i.e. *arg*(*COMANV*)) and the CAN (i.e. ||*COMANV*||).
4. Compute the saccade amplitude from CAD and CAN.
5. Generate the saccade

### CAD and CAN from EMD

The collision avoidance algorithm has been designed on the basis of geometrical optic flow and operates successfully on this basis. However, the properties of the optic flow, as measured by EMDs, differ considerably from the geometrical optic flow. Several model variants of EMDs have been developed (e.g. [28–30]). We used a rather simple EMD model version (similar to [31, 32]) in this study, because we wanted to test whether collision avoidance can already be accomplished by the basic correlation-type motion detection mechanisms with as few model parameters as possible. The drawback with EMDs, at least from the perspective of velocity estimation, is that their responses do not only depend on the velocity of the retinal images, but also on their contrast and other textural properties [33, 34]. Therefore, it is not clear in advance whether the collision avoidance algorithm, as described above and being successful based on geometrical optic flow, will also work with optic-flow estimates based on EMDs. The collision avoidance algorithm based on EMDs will be tested in two steps. In this section, we will assess to what extent the COMAMV derived from EMD measurements matches the COMAMV based on geometrical optic flow. In the next section, we will test the collision avoidance algorithm equipped with EMDs under closed-loop conditions.

**Fig 5:**
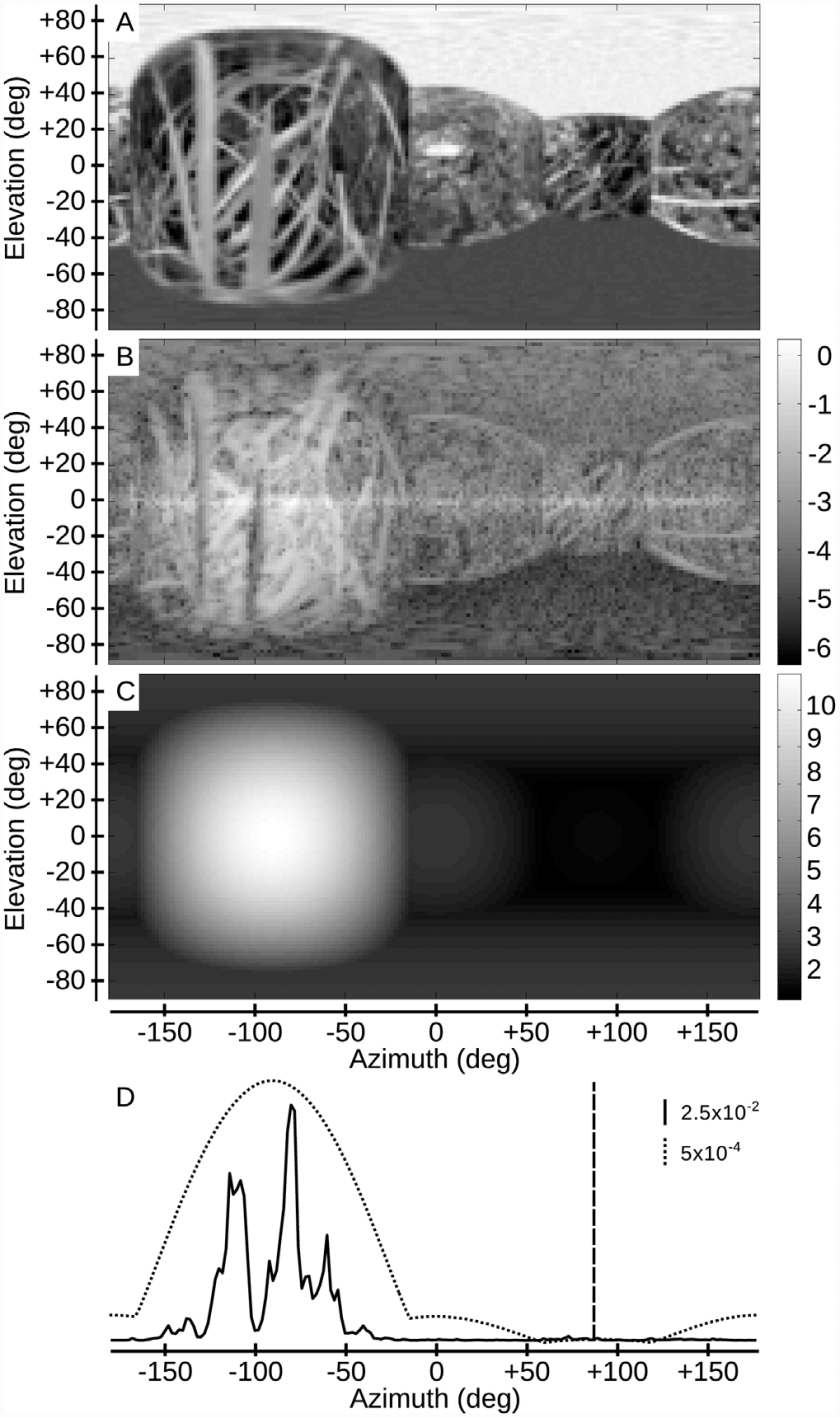
EMD responses and nearness map. A) Panoramic view of the environment, consisting of a cubic box covered with a natural grass texture, from the location where the nearness map was computed (front is azimuth 0°). B) log-scaled nearness map computed on the basis of EMD responses. C) Nearness map at the same location computed from the geometrical optic flow. D) Vertically integrated nearness map extracted respectively from EMD responses (solid line) and geometrical optic flow (dotted line). The vertical dashed line shows the CAD computed from the vertically integrated nearness map based on EMD responses. The direction matches the one computed with geometrical optic flow.

**Fig 6:**
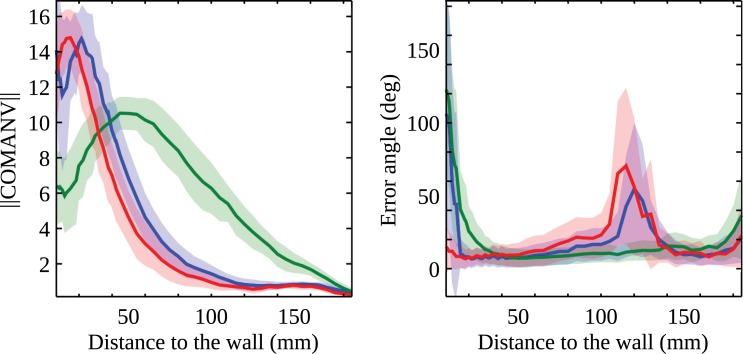
COMANV versus wall distance in a cubic box. The box (height: 390mm) was covered with random checkerboard patterns of either 1mm (blue) or 4mm (green). Red: the box had a height of 3900mm and was covered with a 1mm random checkerboard pattern. Left panel shows the norm of the COMANV computed on the basis of EMD responses. Right panel shows the angle between the COMANV computed from EMD responses and the control based on geometrical optic flow. Thick lines and shaded area represent the mean and the standard deviation, respectively, computed at a given distance from the wall.

As the first essential step, the relative nearness map is extracted from EMD responses. The texture dependence of the EMD measurements is somewhat reduced by spatial averaging along the elevation of the visual field (see above). The consequences of this averaging are shown in Fig 5 for an exemplary simulation (see also S7 Fig). The agent performed a translation inside a box covered with natural images of grass. The relative nearness map obtained from EMDs does not only depend on the geometrical nearness, but also on the texture of the wall (Fig 5). Although integration along elevation reduces the pattern dependence to some extent, the integrated relative nearness map still contains “fake holes” (e.g. those that result from extended vertical contrast borders; Fig 5). These “fake holes” may mislead the agent when looking for relative nearness lower than a certain threshold. As the second step of the collision avoidance algorithm, the COMANV and the CAD have to be computed from the EMD-based relative nearness map. Ideally, the CAD based on EMDs should coincide with the one determined from the geometrical optic flow. The CADs determined in both ways are the same for the example shown in Fig 5. To assess whether this finding also generalizes to other environments, the simulations were extended to cubic boxes with the agent translating parallel to one wall of the box from different starting positions. The angle between the CAD based on EMDs and the one based on geometrical optic flow were computed for every starting position. Fig 6 shows that the CADs based on geometrical optic flow are similar to the CADs based on EMDs if the agent is not too close to the wall and not too close to the center of the box (S4 Fig). Moreover, the higher the walls of the box are, the more CADs determined in the two ways coincide (Fig 6).

The collision avoidance algorithm requires an increasing length of the COMANV with an increasing relative nearness to objects, in order to provide a good estimate of the CAN. The variation of the length of the COMANV with the distance to the wall has been studied with geometrical optic flow in a cubic box. The same environment has been tested with EMDs, but with several different patterns. Similar to the simulation of CAD, the length of the COMANV has been computed for several points in the corridor. Fig 6 shows the dependence of the CAN on the nearness to the wall. As expected, the wall texture changes the CAN, as does the nearness to the wall. However, the CAN is still an increasing function of the nearness to the wall as long as the agent is not too close to the wall (S4 Fig and S6 Fig). Therefore, the norm can be used as a reasonable estimate of CAN in this range.

### Collision avoidance with EMD

As shown above, information about the three-dimensional shape of the environment around the agent derived from EMD responses leads to appropriate CADs and a reliable estimate of CAN, as long as the agent is not too close to an obstacle, such as the wall of a flight arena. These results were obtained in open-loop simulations. Since EMDs use temporal filters, their responses also depend to some extent on the signal history. The time constant of the low-pass filter in one of the EMD branches is 35ms, i.e. in roughly the same range as the time between subsequent saccades of insects (20 to 100ms in flight arenas [8, 14] and 50 ms in our simulation). Thus, the EMD response during a given intersaccade also depends on the signals generated during the previous saccade, resulting in a somehow disturbed nearness map. Taking all this into account, open-loop simulations do not allow the collision avoidance performance under closed-loop conditions to be predicted.

**Fig 7:**
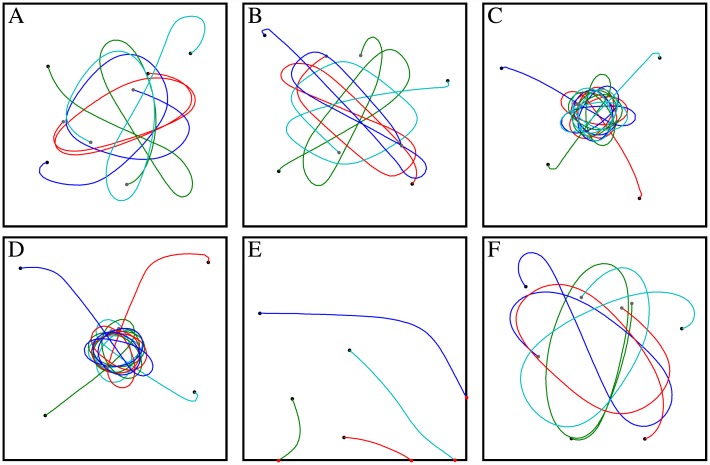
Trajectories of the agent with a collision avoidance system based on EMDs in a box (40 × 40 × 40cm) covered with different patterns (seen from above). Trajectories with four different starting positions are shown (see S12 Fig for different starting position). The simulation time was 10sec or until the agent crashed. Walls of the box are covered with a natural pattern (A), a 1mm random checkerboard (B), a 4mm random checkerboard (C), an 8mm random checkerboard (D), a 35mm random checkerboard (E), and a random pattern with 1/*f* statistic (F). The gain and the threshold of the weighting function was 2 and 4, respectively, for all cases.

A relatively simple and commonly used environment for experiments on collision avoidance behavior of insects are cubic or cylindrical flight arenas [8, 35–37]. In such an environment, the agent has to avoid only the wall. Thus, the task is easier to accomplish than if objects are also present. Fig 7 shows closed-loop simulations in boxes covered with six different wall patterns (see also S8 Fig). The agent is able to avoid collisions for all wall pattern conditions except the random pattern with relatively large (35mm) pixels. However, the area covered by the flight trajectories varies tremendously with the pattern. The saccade amplitude depends on the gain and the threshold, which parameterize the sigmoid function of the CAN. These parameters have been kept constant for the different pattern conditions. By adjusting the threshold and the gain individually for each pattern condition, collision avoidance may be successfully performed by the agent, as long as the CAN increases with the nearness to objects and the CAD points away from obstacles (S4 Fig and S5 Fig).

**Fig 8:**
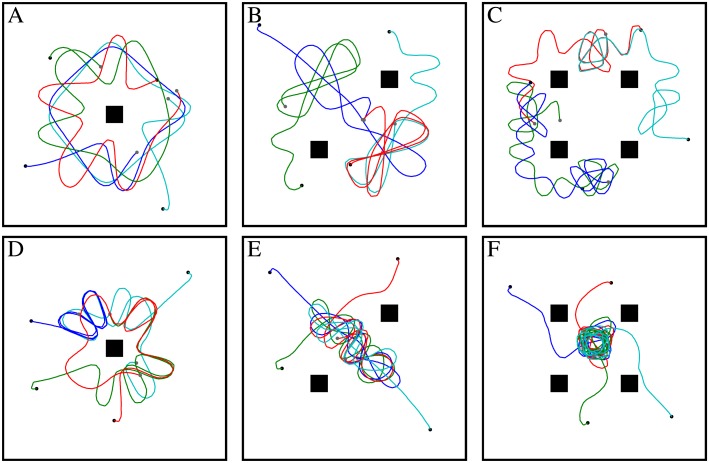
Trajectories of the agent with a collision avoidance system based on EMDs in a box (40 × 40 × 40cm) containing up to four objects and covered with different patterns (seen from above). The pattern on the objects and wall were 1mm and 4mm random checkerboards for the top and bottom panels, respectively. A, D) One object in the center of the box. B, E) Two objects on one diagonal. C, F) Four objects on the diagonals. The objects were vertical bars with a quadratic base with a side length of 3cm and a height of 40cm. The gain and the threshold of the weighting function was 2 and 4, respectively, for all cases.

Until now, we have used a rather simple environment compared to those experienced by an agent under more natural conditions. Objects were added to the flight box to increase the complexity of the collision avoidance task. The objects had the same sizes, shapes and textures. They were camouflaged, i.e. covered with similar patterns to the background, to increase the difficulty for the collision avoidance algorithm. Such situations occur frequently in nature, e.g. when a particular leaf is located in front of similar leaves. To discriminate such an object and to avoid a collision with it, relative motion on the eyes induced by self-motion of the animal and, thus, the relative nearness to the object as obtained from optic flow is the only cue available. Up to four objects were inserted into the box and covered with the same pattern as the walls. The agent was able to avoid collisions successfully, even in the box with four objects (Fig 8, S9 Fig and S10 Fig). However, collisions were observed in boxes containing two and four objects each covered with a 4mm random checkerboard pattern (S10 Fig).

### Collision avoidance with a goal direction

**Fig 9:**
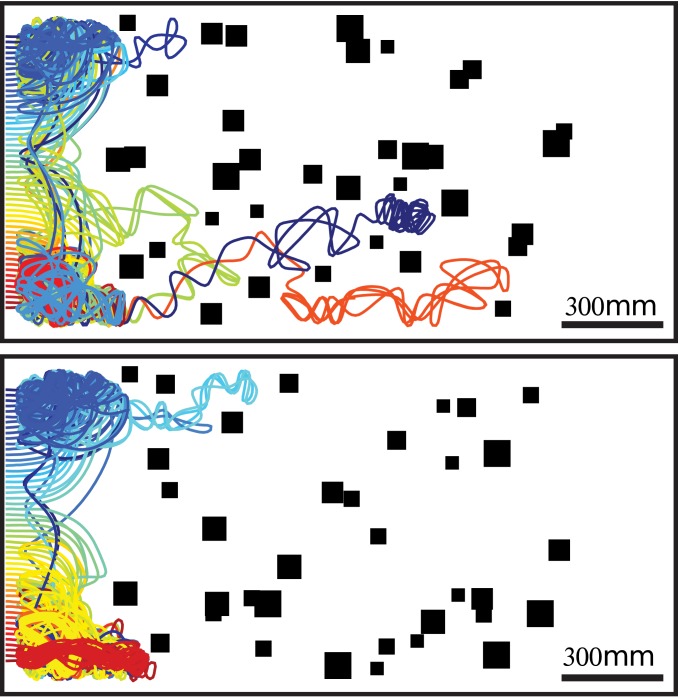
Trajectories of the agent equipped with an EMD-based collision avoidance system in two different cluttered environments with objects and the walls covered by 1mm random checkerboard patterns (seen from above). Fifty-one starting positions were tested, and simulations were run for 100sec or until the agent crashed. The trajectories are color-coded depending on their starting position. Objects are indicated by filled black squares. The gain and the threshold of the weighting function was 2 and 4, respectively, for all cases. (see also S1 Video).

Agents in natural environments may have to face even more complex situations than those tested so far, such as avoiding collisions in a cluttered environment with many objects. A forest is an example which contains many trees, i.e. many objects to avoid. Two different artificial environments with 35 randomly placed objects have been used to test the collision avoidance performance in cluttered environments. Again the objects were camouflaged with the same texture that covered the floor and the confinement of the environment. The agent tended to stay in a relatively small area of the environment where the walls were sufficiently distant (Fig 9). Hence, an agent equipped with only a collision avoidance algorithm did not travel through the artificial forest.

**Fig 10:**
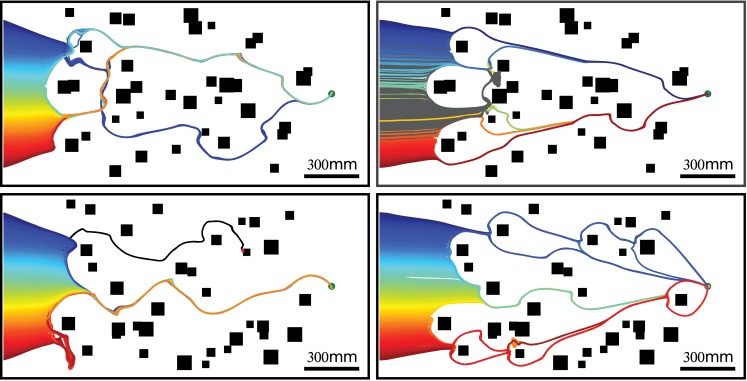
Trajectories of the agent equipped with an EMD-based collision avoidance system, but also relying on the goal direction, in two different cluttered environments with objects and walls covered by 1mm (right column) or 4mm (left column) random checkerboard patterns. The goal is indicated by the green dot. Two hundred one starting positions were tested and simulations were run either for 100sec (gray lines, i.e. dead-end), until the goal was reached (colored lines) or until a crash occurred (black lines). Note that the individual trajectories converge to only a small number of distinct routes. Apart from taking the goal direction into account, the simulations, parameters and environments are identical to those used for Fig 9. (see also S2 Video,S3 Video).

This task was only accomplished if the collision avoidance algorithm was slightly modified to support a goal direction. The saccade amplitude, so far, was the result of a compromise, based on the CAN, between the CAD and the tendency to keep the previous direction of motion. If this direction was replaced by the direction toward a goal, the saccade amplitude became a compromise between the CAD and the goal direction, depending on the CAN. When the CAN was below the threshold, as parameterized by the sigmoid function of the CAN, the saccade amplitude is mainly driven by the goal direction. By contrast, when the CAN is higher than this threshold, saccades would be mainly driven by the CAD. The significance of the CAN could clearly be seen for trajectories close to objects. Far from the object, the agent moved toward the goal, but when it came close to the object, saccade amplitudes tended to be driven by the collision avoidance algorithm, pushing the agent in the opposite direction (Fig 10). When the goal was located at the other end of the corridor, the agent was efficiently, i.e. without making many detours, and reliably, i.e. with a low rate of collisions, able to reach the goal (Fig 10,S2 Text,S11 Fig).

### Route similarity in cluttered environments

The number of different trajectories close to the goal location in cluttered environments is much lower than the number of starting conditions. Therefore, agents, starting from different locations, but heading towards the same goal location, have trajectories converging to similar routes. This behavior is not only a consequence of the walls confining the cluttered environment. Indeed, a similar behavior is observed in a cluttered environment without confining outer walls (S11 Fig). In order to classify the similarity of the different trajectories, each trajectory was first simplified into a sequence based on the position of the agent relative to the objects in the environment. Trajectories sharing the same sequence formed one class, i.e. a route [see material and methods]. In the first (resp. second) cluttered environment, 8 (resp. 11) and 4 (resp. 3) distinct routes were found for objects and walls covered with 1mm and 4mm random checkerboard patterns, respectively (S12 Fig, S13 Fig, S14 Fig, S15 Fig)

**Fig 11:**
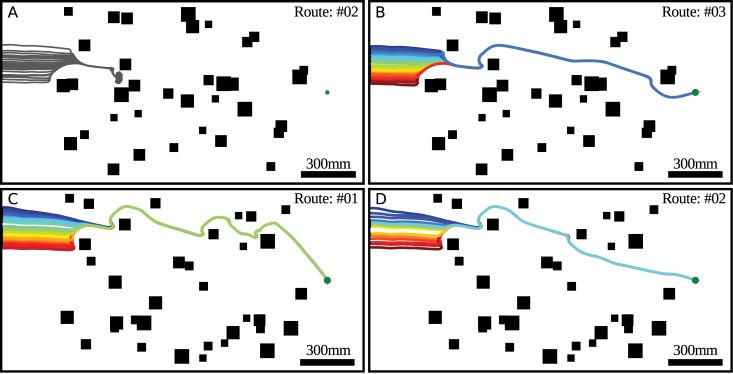
A selection of the routes shown in Fig 10) for the two environments. Although the area of starting positions greatly overlap for a given environment, the trajectories converge on two different routes (compare A with B, and C with D). The simulations, parameters and environments are identical to those used for the right panel of Fig 9.

The routes followed by the agent may be determined by its starting position, i.e. neighboring starting positions may lead to the same route. Indeed, when an agent approaches an object from the right (resp. left), it tends to avoid it by a left (resp. right) turn. This “decision” will be taken for every obstacle along the trajectory taken by the agent, but each “decision” depends sensibly on the position of the agent relative to the object and the goal location. Therefore, the route followed by an agent may be sensitive to the starting position. Fig 11 shows that neighboring starting locations may lead to different routes.

The number of different routes close to the goal location is lower than the number of possible routes in a given environment. This indicates that, on the one hand, routes starting at different locations tend to converge into common routes and, on the other hand, different routes may share similar parts, i.e. sub-routes. As a measure of similarity between routes, the number of single sequence elements differing between two routes was used. Routes may be very similar to each other with less than five different single sequence elements (e.g. compare route #8 and route #10 in S14 Fig). Different routes, therefore, share similar sub-routes. This indicates that a rather small number of locations exist where the agent “decides” to take a particular sub-route, e.g. avoid an object towards the left or the right, respectively.

**Fig 12:**
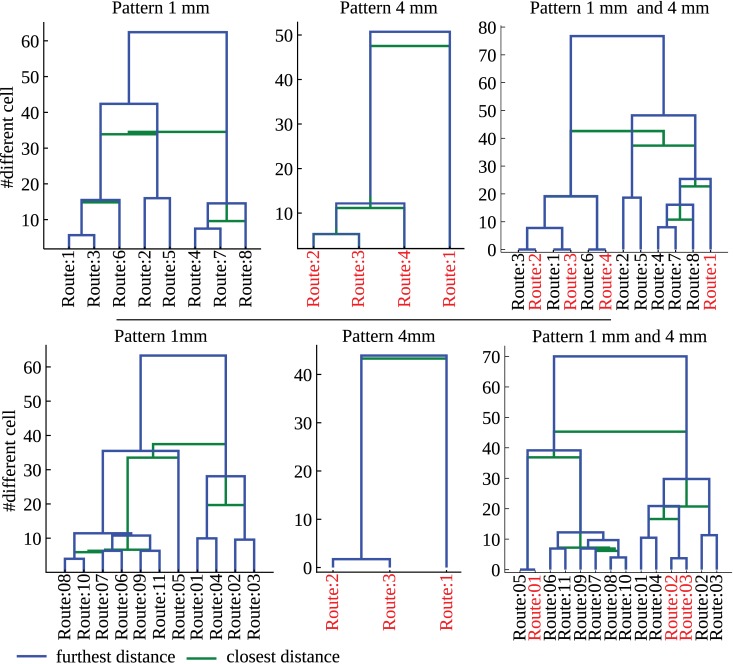
Dendrogramm of route similarity for the two different cluttered environments, top and bottom row, respectively. The routes followed by the agent (see Fig 9) are characterized by a cell sequence. Here, each cell is a triangle formed by neighboring objects. The route similarity is defined by the number of cells not shared by routes. First and second columns, path-similarity for 1mm and 4mm random checkerboard patterns, respectively. Third column, path-similarity across patterns. Note that identical routes are found for different patterns: for example, route #3 and route #2 in the first environment (top row) are identical to those for the environment covered by 1 mm and 4 mm random checkerboards, respectively. The routes are shown in S12 Fig,S13 Fig,S14 Fig,S15 Fig.

The collision avoidance algorithm is affected by the pattern covering the walls and the objects in the environment. This dependency may lead to different routes. Therefore, routes obtained in an environment with given object locations have been compared after changing the texture of the environment to pinpoint texture-dependent effects. Interestingly, certain classes of routes are indeed the same for the different patterns, e.g. the second route for a 1mm random checkerboard texture matches the third route for a 4mm random checkerboard texture (Fig 12). Three routes (resp. one) out of the four (resp. three) routes for the 4mm random checkerboard textures are indeed found also for the 1mm random checkerboard textures covering the first (resp. second) environment. This finding indicates that, despite pronounced pattern effects resulting from the properties of EMDs, the performance of the collision avoidance algorithm is, on the whole, quite stable and, to a large extent, depends on the spatial structure of the environment.

## Discussion

We developed a model of collision avoidance based on correlation-type elementary motion detectors (EMDs), which accounts for the fundamental abilities of insects to avoid collisions with obstacles in simple and also complex cluttered environments. The model has been developed with geometrical optic flow, showing that apparent motion as the only source of information about the depth structure of the environment is sufficient to accomplish collision avoidance tasks. The transfer from geometrical optic flow to bio-inspired correlation-type EMDs has been shown to be successful in a range of environments. Moreover, when the collision avoidance algorithm is coupled to a goal direction, the coupling between goal-directed behavior and collision avoidance allowed the agent to move through cluttered environments, even if the objects, floor and the background were covered with the same texture. Interestingly, the trajectories traveled in the cluttered environment were very similar, irrespective of the starting condition.

In the following, we discuss three key aspects of this work: (1) the duration of the intersaccades and their shape and, thus, the conditions under which spatial information about the environment can be obtained; (2) the nearness measurements obtained from EMDs; and (3) how the convergence of individual trajectories into a small number of routes can arise from collision avoidance while the agent is heading towards a goal.

### Changes in intersaccadic translation direction

The assumptions underlying our algorithm to extract a nearness map from optic flow are: (i) a spherical eye, (ii) a translation phase combining several directions of motion (i.e. a mixture of different forward and sideways motions), and (iii) all movements of the agent take place in the null elevation plane. The second assumption is required in order to average out the characteristic singularities in translational optic-flow fields, i.e. the FOE and the FOC, by integrating the optic-flow amplitudes obtained during translations in slightly different directions (i.e. a mixture of forward and sideways motions). Indeed, the direction of translational movements between saccades of flying insects is not always constant with respect to the orientation of the body long axis. This means that the relationship between the forward and sideways motion components may change systematically even between two consecutive saccades. Extreme examples in this regard are shown in Fig.3 of [8]. However, more moderate continual changes in the ratio between forward and sideways translational components occur, as a consequence of inertia, after virtually all saccades, with the strength of these changes depending on saccade amplitude [8, 14]. Therefore, flying insects could average the optic flow generated on the eyes during these continual intersaccadic changes in flight direction and then extract the relative nearness to determine the direction and amplitude of the next saccade. However, as a consequence of the inevitable time constants of the motion detection system, the EMD responses following a saccade might also be affected by the rotational optic flow of the previous saccade. Although the rotational part of the optic flow could, in principle, be removed if the angular velocity of the agent was known, this transformation would not be straightforward on the basis of motion measurements based on EMDs [38], given their dependency on the texture of the environment.

The integration of the optic-flow amplitudes along intersaccades is also important to decrease the dependency on the texture in the environment characteristic of EMD responses. For simplicity, the intersaccade duration has been kept constant in our simulations, although in free-flying flies, it was found to vary from 20 to 100ms [8, 14]. By increasing the duration of an intersaccade, the dependency on the texture in the environment can be further decreased if the integration time is increased accordingly. An increase in integration time has similar effects to increasing the extent of spatial integration along the direction of motion [39]. However, the longer the duration of the intersaccadic translation is, the more blurred the relative points of nearness are, as shown in Fig 2. On the other hand, the intersaccade duration might be linked to the collision avoidance necessity. Indeed, collision avoidance may be unnecessary when no obstacles are encountered, as shown by the closed-loop simulations of goal-oriented behavior (Fig 9 and Fig 10). If the collision avoidance necessity is low, long intersaccades are possible. However, if the collision avoidance necessity is high, short intersaccades followed by an evasive turn are required.

The third assumption of the algorithm of nearness calculation, i.e. that the agent only moves in the null elevation plane, is certainly not exactly satisfied in free-flying insects. However, during most flight manoeuvres, changes in height occur to a much smaller extent than changes in the horizontal plane. Nonetheless, if an agent moves in another direction than in the null elevation plane and estimates the nearness with our algorithm, its estimation will have an error proportional to the upward component (S1 Text). As long as this component is small, the estimated nearness map will not be strongly affected. We are currently investigating how our algorithm for nearness estimation can be extended to arbitrary movements in three dimensions.

### Nearness from measured optic flow

We have shown that relative nearness can be extracted from geometrical optic flow. However, if the nearness algorithm receives its input from correlation-type EMDs, complications arise from the dependence of the EMD responses on (i) the contrast of the stimulus pattern, (ii) its spatial frequency content, and (iii) the fact that it is not related to velocity in a linear way, but first increases, reaches an optimum value and then decreases again [40]. Indeed, these dependencies are somehow reflected in the extracted relative nearness (see, for example, Fig 5D). The EMD has a quadratic response dependence on contrast if no additional nonlinearities are inserted in its input lines [16]. Indeed, it has been recently shown that the norm of the EMD response is correlated to the nearness times the local contrast or, in other words, EMDs have been shown to respond best to the contrast contours of nearby objects (see also, Fig 5) [41]. The dependency on contrast can, in principle, be reduced by applying a nonlinearity before the multiplication stage of an EMD [28, 29, 33]. Nonlinearities tend, however, to complicate the mathematical analysis of the EMD response. Therefore, we have chosen to use a simple EMD version. Moreover, we wanted to test how well the collision avoidance algorithm performs on the basis of the basic correlation-type motion detection mechanism with as few model parameters as possible.

The average output of an EMD depends on the temporal frequency of a motion stimulus (i.e. the ratio of angular velocity and wavelength of its spatial Fourier components) rather than its real velocity [33, 34]. To extract the real angular velocity from EMD responses, the dependency on temporal frequency needs to be reduced. Plett et al. suggested using the spatial power spectrum of a panorama to extract the angular velocity from EMD responses during rotation [38]. However this transformation is not suitable for translation, because the spatial frequency content of the panorama is not related unambiguously to the temporal frequency observed during translation. Therefore, it is not easily possible to compensate for these dependencies.

Nearness extracted from the nonlinear, but monotonic response range of the EMDs may lead to a distorted representation of the depth-structure of the environment. Nevertheless, larger EMD responses still correspond to greater nearness. Ambiguous nearness estimates will arise for the nonlinear and non-monotonic, i.e. the ambiguous, response range of the EMD. Therefore, close objects leading to large retinal velocities might be mistaken for far objects. In the context of nearness extraction during translation, this problem will arise for objects on the lateral side of the agent. To avoid this problem, an agent has two possibilities: reduce its translational speed or extract nearness information only in the more frontal parts of the visual field. The agent in our simulations was moving at a relatively slow speed. Therefore, this ambiguity was not observed. Moreover, flying insects have been concluded to use the frontal part of their visual field to compute saccade amplitudes (flies, [14]) and to reduce their flight speed when the clearance to objects in the environment gets small (e.g. bumblebees [42]; flies: [14]). It is, however, unclear how flying insects compute the CAD only taking into account motion measurements in the frontal part of the visual field.

A robot does not need to estimate optic flow with EMDs. The estimation can be carried out with image-based methods, e.g. the Lucas Kanade algorithm [6], or event-based methods [43]. Image-based methods are, however, time-consuming and, therefore, are not really suitable for applications in real-time. However, event-based flow-field detectors are fast and reliable. Our algorithm to extract nearness from optic flow is only local, i.e. it only uses the optic-flow vector in a given viewing direction to compute the relative nearness in this direction. Therefore, our collision avoidance algorithm could be easily coupled with EMDs to determine the nearness around the robot in real-time as a cheap alternative to a 3D laser rangefinder.

### Goal-directed collision avoidance and route following

Both biological and technical agents often need to reach a goal, e.g. their nest in the case of many insects or a charging station in the case of a robot, without colliding with the objects along their trajectory. This goal direction in a real world could be provided by path integration and visual navigation [44] or, in the case of a technical agent, by GPS. Therefore, when the agent is in cluttered environments, it needs to somehow integrate the goal direction and the collision avoidance direction. Our collision avoidance algorithm has been shown to support a goal direction by using CAN, leading to a behavior that represents a kind of compromise between collision avoidance and reaching the goal. Interestingly, the trajectories of the agent, even in complex cluttered environments, tend to converge on a limited number of distinct routes largely independent of the starting position (see Fig 10) when coupled to a goal direction. The appearance of routes is not a unique property of our collision avoidance algorithm. Similar trajectories are also followed by an agent with different control strategies and different collision avoidance algorithms (e.g. see Fig.6. in [19], and Fig.4. in [23]). Ants perform in a similar way and also follow similar trajectories in cluttered environments when returning to their nest (e.g. [45–48]). We could show that this type of behavior can be explained in a relatively simple way by combining our local collision avoidance algorithm only with an overall goal direction. By contrast, the similarities of trajectories of ants have often been interpreted within the conceptual framework of a route-following paradigm. According to this paradigm, the agent is assumed to store local information along the trajectory during an outbound run (e.g. leaving the nest), which will be used to determine the direction to follow during an inbound run (e.g. returning to the nest) [49, 50]. In our simulations, we observed that trajectories which may be interpreted as resulting from route-following could, alternatively, arise just from a collision avoidance algorithm coupled to a goal direction. Therefore, part of the route-following behavior observed in insects could be a consequence of a collision avoidance algorithm. Hence, the route-following direction does not need to be determined at every point along the trajectory, but its determination may be sparsely spaced.

The routes to the goal followed by our agent depend on the starting location, i.e. neighboring starting locations may lead to different routes (Fig 11). The same behavior has been observed in ants [48]. However, the different routes are not equivalent in term of efficiency. Indeed, in Fig 11A and 11B, the route #2 is dead-end, and route #3 reaches the goal. An agent may need to use the most efficient route, i.e. add waypoints in the environment indicating which route to follow. The routes #2 and #3 (resp. #1 and #2) shown in Fig 11, for example, could be merged by adding only one waypoint just where the two routes emerge. Therefore, insects may place a small number of well chosen waypoints in the environment to prevent the dead-end problem observed in our simulations 10 and possibly to select the most efficient routes (S2 Fig) without requiring a large memory.

## Materials and Methods

The simulations have been inspired by the flight and gaze strategies of flies and other insects. The trajectories have, thus, been separated into saccadic and intersaccadic phases. The intersaccade, i.e. a translation of 50ms at 0.1ms^−1^, is used to gather information about the depth-structure of the environment from optic-flow measurements and to provide a CAD. The CAD controls the amplitude of the saccade following the translation phase. The yaw velocity of the saccade is computed according to a Gaussian velocity profile. This profile fits the experimentally determined template of saccades [8, 31]. Therefore, the saccade duration and the peak yaw velocity are given by the template and the saccade amplitude. Closed-loop simulations were performed in environments of different complexity, which were covered with different textures. The simulations were mainly written in MATLAB, with part of the code written in C for computational efficiency, and run at a sampling rate of 1kHz.

### The simulation environment

The environments used in our simulations have been inspired by previous behavioral experiments on flies and bees. Schilstra and van Hateren used a cubic box with an edge length of 40cm [8, 9]. This box was covered with natural images on its side walls and with a black/gray and a white/gray irregular pattern on the floor and the ceiling, respectively. The side walls of the box were covered alternatively by 1mm, 8mm or 35mm black and white random checkerboard patterns to investigate the effect of the texture in another set of simulations. Simulations were also done in boxes with one, two or four obstacles covered with 1mm black and white random checkerboard patterns to make the collision avoidance task more difficult. The obstacles had the same height as the box and a square cross-section with an edge length of 30mm. The wall of the box had the same texture as the objects. The obstacles were placed at positions as shown in Fig 8; they were vertical bars of 40cm height and a quadratic base with a side length of 3cm.

Cluttered environments with 35 obstacles of different sizes were used in another set of simulations. Every wall in the environment (object sides and corridor walls) was covered by either a 1 mm or a 4 mm random checkerboard pattern. The obstacles had a square base and a height five times their side length, and were randomly positioned in the inner part of a 2000 × 1000 × 400mm box. Two environments were selected based on the homogeneity of the obstacle positions. The first environment was composed of five objects with an edge length of 80mm, five with an edge length of 72mm, ten with an edge length of 64mm, five with an edge length of 56mm, five with an edge length of 48mm, and five with an edge length of 40mm. The second environment was composed of the same number of objects, except that ten objects with an edge length of 72mm and five objects with an edge length of 64mm were used.

### Visual system and motion detection

Once the environment had been created, a panoramic view from any position within the environment could be generated and the distance to objects determined. The set of vertices X,Y,Z that define an object, as well as the floor and the ceiling, were translated to the current position of the agent. An environment map was rendered using OpenGL. The input image was sampled by Gaussian-shaped spatial low-pass filters (*σ* = 2°). The output of these filters formed the input to the photoreceptors that were equally spaced at 2° along the elevation and azimuth of the eye. The array of photoreceptors formed a rectangular grid in the cylindrical projection with 91rows and 181columns. The temporal properties of the peripheral visual system was modeled as a temporal filter with a kernel that was derived from an electrophysiological analysis of the responses of second-order visual interneurons in the fly visual system to white-noise brightness fluctuations [51, 52]. The filter kernel is a kind of temporal band-pass filter with a DC component (for a formal description, see [31]). The outputs were, furthermore, filtered with a first-order temporal high-pass filter (time constant 20ms) to remove the DC component. The filtered outputs of neighboring elements were fed into elementary motion detectors of the correlation type with a first-order temporal low-pass filter (time constant 35ms) in one of its branches. Each local movement detector consisted of two mirror-symmetrical subunits. In each subunit, the low-pass filtered signal of one input channel was multiplied with the high-pass filtered signal of the neighboring input channel. Elementary motion detector signals depend on the scenery (e.g. [33]). Therefore, a benchmark of optic-flow measurement independent of scenery was necessary. The optic flow can be computed when the self-motion of the agent and the nearness to objects is known. In a virtual environment, both pieces of information are accessible. The set of vertices X,Y,Z, that defines the object were translated to the current position of the agent. Then, the nearest point on the retina for each viewing direction was extracted. The geometrical optic flow was then computed from the nearness and the self-motion [24], giving a motion measurement independent of the texture of the scenery.

### From EMD responses to nearness estimates

The optic flow experienced during translation is linked to the nearness of the agent to objects in the environment and its self-motion. Assuming that the agent moves in the null elevation plane and uses a spherical eye, it can be shown that the relative nearness (*vμ*) is linked to a retinopically modified norm of the optic flow:

$${\left(v\mu (\epsilon ,\phi )\right)}^{2}=OF{\left(\epsilon ,\phi \right)}_{\hat{\phi }}^{2}+\frac{OF{\left(\epsilon ,\phi \right)}_{\hat{\epsilon }}^{2}}{\sin^{2}(\epsilon )}$$ (1)

where *v* is the speed, *μ* is the nearness to the object in the viewing direction (*ϵ*, *ϕ*), *ϵ* the elevation, *ϕ* the azimuth, and *OF* the optic-flow vector. This function holds as long as the elevation is not zero, but a similar equation can be used for null elevation (S1 Text). The relative nearness cannot be computed at the FOE or the FOC due to the singularity, i.e. absence of apparent motion, in the flow field. To remove these singularities, the flow fields resulting in two different motion directions at the same location in the environment might be averaged. However, insects are unlikely to fly twice at the same position in the environment. However, they can fly subsequently in two different directions at two nearby points in space. The nearness at those points will be almost equal as long as the distance between these points is relatively small. Therefore, the agent performed a translation, composed of 50 segments with different motion directions. The motion direction of each segment followed a normal distribution centered at zero with a standard deviation of 18° (Fig 1 part 1). The translation is thought to correspond to an intersaccade of insect flight. A stack of optic-flow fields along the time, i.e. during the intersaccade, was gathered (Fig 1 part 2). Although it is possible to compute the nearness for each optic-flow field and then integrate the nearness over time, we used an alternative, but equivalent approach. The optic-flow fields were squared and integrated over time (Fig 1 part 3). Then, the integrated squared optic flow was used to compute the nearness map of the environment (Fig 1 part 4).

When the optic-flow field is estimated by EMDs, the estimations also depend on the motion history due to the temporal filters in the EMD. The saccade preceding the translation, therefore, interferes with the optic-flow measurements during the intersaccadic interval. This effect decreases over time. Therefore, the optic-flow field was not integrated during the entire intersaccadic phase, but only for the last five segments (i.e. last 5 ms).

### Nearness and collision avoidance

Once the nearness map is known, it is an obvious strategy to avoid collisions by moving away from the maximum nearness value. However, the nearness map derived from the EMDs also depends on pattern properties. Thus, the nearness map was averaged along the elevation, giving the average nearness for a given azimuth and, thus, reducing the texture dependence (Fig 1 part 5). Each of these averaged nearness values could be represented by a vector in polar coordinates, where the norm of the vector is the averaged nearness and its argument the azimuth. The sum of these vectors points towards the average direction of close objects in the environment when the effect of the pattern on the EMD responses is sufficiently averaged out. Thus, the opposite of this vector will point away from the closest object and, thus, is selected as the motion direction of the agent in order to avoid a collision (Fig 1 part 6).

Moreover, the length of the vector increases with the nearness to objects and the apparent size of the object. Thus, its length can be used as a measure of the collision avoidance necessity. This measure drives the state of the animal between “collision avoidance” and “move in the previous direction” according to the following equation:

$$\begin{array}{c} \begin{array}{cc} \gamma & =W\left(∥COMANV∥\right)\text{arg}\left(COMANV\right)+\left(1-W\right)\left(\alpha +\sigma \right)\\ W\left(∥COMANV∥\right) & =\frac{1}{1+{\left(\frac{∥COMANV∥}{n_{0}}\right)}^{-g}} \end{array} \end{array}$$ (2)

where, *COMANV* is the vectorial sum of the vertically integrated nearness values, *W* is the weighting function based on the norm of the COMANV, *α* the goal direction and *σ* is a goal direction noise. The weighting function used in the simulation is a sigmoid function, which is driven by a gain *g* and a threshold *n*_0_.

The goal direction has been fixed to zero for the simulation in boxes. The agent, thus, continues moving straight, i.e. a saccade with null amplitude, when the CAN is zero. In other simulations, the goal direction is different from zero. The agent then performs a saccade amplitude driven by the goal direction when the CAN is zero.

### Cluster of trajectories and route similarities

Even when the starting position of different runs of the agent differ, the trajectories that are taken by the agent in a given cluttered environment tend to converge to similar routes. When approaching an obstacle, an agent may avoid it by a left or right turn, leading to either of two different routes. Therefore, the obstacles are the main factors affecting the overall structure of the trajectories and, thus, their similarity. In order to cluster trajectories into routes, a triangular meshing (Delaunay triangulation [53, p. 513-529]) of the environment was calculated with the nodes of the meshing corresponding to the center of mass of the obstacles. The meshing is, thus, composed of triangular cells formed by three neighboring obstacles. The cells in the meshing do not overlap. A trajectory of the agent crosses a succession of triangular cells and can, therefore, be associated to a sequence. Here, each element in the sequence represents a given cell in the meshing, i.e. a given region of the environment. The agent may visit a region more than once, by making detours or oscillating between two neighboring cells. The sequence was simplified in order to remove multiple visit by suppressing subsequences between identical sequence elements.

Once each trajectory has been associated to a sequence of cell occupancy, the trajectories sharing exactly the same sequence were attributed to a cluster, i.e. a route. Each route corresponds, therefore, to a unique sequence. Different routes may be similar. To quantify the similarity between routes, the number of single sequence elements, i.e. a cell, not shared by two routes was used. This measure of route similarity is similar to the Hamming distance [54] between route sequences.

## Supporting Information

S1 Text From optic-flow to relative nearness. Advantage of a spherical eye: Derivation of a link between relative nearness and optic-flow for motion constrained in the null elevation plane of the agent. Error on relative nearness: Study the error on the extraction of relative nearness when the motion deviates from the constrain. Finally the limitations of a cylindrical eye compare to a spherical one are reported.
(PDF)

S2 Text Performance of the algorithm in cluttered environments. The performance is quantified in term of its reliability, i.e. the percentage of trajectories with a collision occurring before the goal is reached, and its efficiency, i.e. the distance traveled needed to reach the goal. The later is compared to the shortest path, and a collision proof path, both derived from the object positions in the environment.
(PDF)

S1 Fig Variation of horizontal and vertical color-coded optic-flow component squared for a motion in the null elevation plane in the center of a sphere. A) Vertical optic-flow component squared. B) Horizontal optic-flow component squared. C) Vertical optic-flow component squared divided by the sine squared of the elevation of the viewing direction, i.e. the retinotopically corrected vertical optic-flow component. The null azimuth corresponds to the motion direction of the agent. Note that the horizontal component and the retinotopically corrected vertical optic-flow component have an antagonistic variation (compare B and C). The color code marks larger optic-flow values with warmer colors, and represents angular velocity in rad.s-1.
(EPS)

S2 Fig Performances of our algorithm based on the distances of travel to reach the goal in a cluttered environment relative to the distance of the shortest trajectories. The performance is the length of the shortest trajectory divided by the length of the trajectory followed by the agent. A, D) Relative distances of travel to reach the goal as a function of the starting position. Trajectories for the environments covered by a 1 mm and 4 mm random checkerboard pattern, respectively, are shown in Fig 10B and 10E) Voronoi trajectories in the environments. C, F) Shortest trajectories in the environments. A, B and C (resp. D, E and F) are for the first (resp. second) environment. Note that the performance is above 60%, except for the trajectory with a dead-end.
(EPS)

S3 Fig Norm of the COMANV in cluttered environment covered with 1 mm (left panels) and 4 mm (right panels) random checkerboard patterns. Top panels: Color-coded norm of the COMANV. Bottom panels: The red line delimits the zone where the weighting function is equal to 0:5. Here, the gain and the threshold of the weighting function are 2 and 4, respectively. Saccade amplitudes are mainly driven by the CAD when the agent is inside a contour (threshold line) containing an object. The contour represents the switch in behavior. Objects are indicated by black squares.
(EPS)

S4 Fig CAD in a cubic box covered with different patterns. The agent moved toward the north wall. The color represents the angle (in degrees) between the CAD and the direction of motion of the agent. Walls of the box are covered with a natural grass pattern (A), a 1 mm random checkerboard (B), a 4 mm random checkerboard (C), an 8 mm random checkerboard (D), a 35 mm random checkerboard (E), and a random pattern with 1 = f statistic (F). Note that the CAD for the 35 mm random checkerboard points in many places toward the wall of the box (E).
(EPS)

S5 Fig Norm of the COMANV in a cubic box covered with different patterns. The agent moved towards the north wall. The color represents the norm of the COMANV, i.e. the collision avoidance necessity. Walls of the box are covered with a natural grass pattern (A), a 1 mm random checkerboard (B), a 4 mm random checkerboard (C), an 8 mm random checkerboard (D), a 35 mm random checkerboard (E), and a random pattern with 1/*f* statistic (F). Note that the CAN is higher close to the wall than at the center of the box. The CAN remains low for the box covered with natural grass pattern in some places close to the wall, due to large leaves in the pattern.
(EPS)

S6 Fig COMANV versus the distance to the closest wall in a cubic box covered with different patterns. Top row shows the norm of the COMANV computed with EMD responses. Bottom row shows the angle between the COMANV computed with EMD responses and the control based on geometrical optic flow. Here *α* is the CAD. Thick lines and shaded area represent the mean and the standard deviation, respectively, computed at a given distance from the wall. Note that the norm of the COMANV is an increasing function of the distance as long as the agent is not too close to the wall.
(EPS)

S7 Fig COMANV versus the distance to the closest object in cluttered environments. Left panels: Average of the error angle between the COMANV and the vector between the agent and the closest object. Right panels: Norm of the COMANV. The averages are computed with a sliding window with a window size corresponding to 10% of the distance to the closest object. A, B) Effect of the height of the closest object for an agent with a field of view (FOV) along the elevation of ±90°, and moving at an altitude of 100mm above the ground. Note that the height of the object does not strongly influence the COMANV. C, D) Effect of the moving altitude of an agent with a FOV along the elevation of ±90°. Note that the error angle far from the object is higher for motion close to the ground than far from the ground. E, F) Effect of the FOV of the agent. The agent moved at an altitude of 100mm above the ground. Note that the norm of the COMANV is strongly affected by the FOV along the elevation. The FOV along the elevation was ±180° for all cases.
(EPS)

S8 Fig Trajectories of the agent with a collision avoidance system based on EMDs in a box (40 × 40 × 40 × cm) covered with different patterns (seen from above). Trajectories with 40 different starting positions are shown. The simulation time was 10sec or until the agent crashed. Walls of the box covered with a natural pattern (A), a 1mm random checkerboard (B), a 4mm random checkerboard (C), an 8mm random checkerboard (D), a 35mm random checkerboard (E), and a random pattern with 1/*f* statistic (F). The simulations, parameters and environments are identical to those used for Fig 7.
(EPS)

S9 Fig Trajectories of the agent with a collision avoidance system based on EMDs in a box (40 × 40 × 40 × cm) containing up to four objects and covered with different patterns (seen from above). The pattern on the objects and walls is a 1mm random checkerboard. Top panels: Goal direction is moving forward. Bottom row: Goal direction is the right wall. A, E) No object in the box. B, F) One object in the center of the box. C, G) Two objects on one diagonal. D, H) Four objects on the diagonals. The objects were vertical bars with a quadratic base with a side length of 3cm and a height of 40cm. Apart from taking the goal direction into account for the bottom panels, the simulations, parameters and environments are identical to those used for the top panels of Fig 8.
(EPS)

S10 Fig Trajectories of the agent with a collision avoidance system based on EMDs in a box (40 × 40 × 40 × cm) containing up to four objects and covered with different patterns (seen from above). The pattern on the objects and walls is a 4mm random checkerboard. Top panels: Goal direction is moving forward. Bottom row: Goal direction is the right wall. A, E) No object in the box. B, F) One object in the center of the box. C, G) Two objects on one diagonal. D, H) Four objects on the diagonals. The objects were vertical bars with a quadratic base with a side length of 3cm and a height of 40cm. Apart from taking the goal direction into account for the bottom panels, the simulations, parameters and environments are identical to those used for the bottom panels of Fig 8.
(EPS)

S11 Fig Trajectories of the agent equipped with an EMD-based collision avoidance system, but also relying on the goal direction in two different cluttered environments without walls. Objects were covered by a 1mm (left column) or 4mm (right column) random checkerboard pattern. The goal was pinpointed in the environments (Green dot). Two hundred one starting positions were tested, and simulations were run either for 100s (gray lines, i.e. dead-end), until the goal was reached (colored lines) or until a crash (black lines). Wall of the corridors of Fig 11 are represented by thick dotted gray lines and objects by filled black squares. The gain was 2 and the threshold 4 for all cases.
(EPS)

S12 Fig The routes, extracted from the trajectories shown in Fig 11, in the first cluttered environment which is covered by 1 mm random checkerboard. Several routes are found for neighboring starting locations, e.g. routes #4, #5 and #6. Certain routes share common subroutea, e.g. the end of route #7 and #8 are similar. The route similarities are shown in Fig 12. Walls of the corridors are represented by thick black lines, and objects by filled black squares. Same colors as Fig 11.
(EPS)

S13 Fig The routes, extracted from the trajectories shown in Fig 11, in the first cluttered environment which is covered by 4mm random checkerboard pattern. Routes #1 and #2 overlap in their starting locations. However, route #2 is shorter than route #1. Note that routes #2, #3 and #4 are identical to routes #3, #1 and #6, respectively, in the environment covered by a 1mm random checkerboard pattern Fig 12. The route similarities are shown in Fig 12. Walls of the corridors are represented by thick black lines, and objects by filled black squares. Same colors as Fig 12.
(EPS)

S14 Fig The routes, extracted from the trajectories shown in Fig 11, in the second cluttered environment which is covered by 1mm random checkerboard pattern. Note that routes #5–11 and routes #1–4 form two distinct classes (see Fig 12). The route similarities are shown in Fig 12. Walls of the corridors are represented by thick black lines, and objects by filled black squares. Same colors as Fig 11.
(EPS)

S15 Fig Routes in the second cluttered environment, which is covered by a 4mm random checkerboard pattern. Routes #2 and #3 are very similar (see Fig 12) and lead to a crash of the agent. The remaining route (route #1) is identical to route #5 in the environment covered by 1mm random checkerboard (S14 Fig). Walls of the corridors are represented by thick black lines, and objects by filled black squares. Same colors as Fig 11.
(EPS)

S1 Video Simulation of agents without goal direction in the second cluttered environment. The trajectories are shown in Fig 9, bottom. Objects and walls are covered by a 1mm random checkerboard pattern. Objects are represented by black squares, agent’s current positions are represented by colored circles and agent’s past positions are represented by a colored line.
(MP4)

S2 Video Simulation of agents with goal direction in the second cluttered environment. The trajectories are shown in Fig 10, bottom right. Apart from taking the goal direction into account, the simulations, parameters and environments are identical to those used for S1 Video.
(MP4)

S3 Video The agent’s view while moving through a cluttered environment. The trajectory is shown in Fig 10, top-left. Objects and walls are covered with a 4mm random checkerboard pattern. Top: Agent’s view. Overlay: The relative points of nearness along the azimuth extracted from EMD responses during the last intersaccade. The goal direction and CAD are indicated by the green and red line, respectively. The length of the red and green line indicate the necessity to follow the CAD or goal direction based on the weighting function. The dotted line is the saccade direction. Bottom: The agent’s position and orientation in the environment. Objects are represented by black squares, agent’s current position and orientation in blue and agent’s past position in gray. The behavioral state of the agent, saccade or intersaccade is indicated by a white circle.
(MP4)
