# Supplementary material for: A Bio-inspired Collision Avoidance Model Based on Spatial Information Derived from Motion Detectors Leads to Common Routes
Source: PLoS Comput Biol. 2015 Nov 19;11(11):e1004339. doi: 10.1371/journal.pcbi.1004339 (PMC4652890; doi:10.1371/journal.pcbi.1004339)
Supplement: S1 Text — Advantage of a spherical eye: Derivation of a link between relative nearness and optic-flow for motion constrained in the null elevation plane of the agent. Error on relative nearness: Study the error on the extraction of relative nearness when the motion deviates from the constrain. Finally the limitations of a cylindrical eye compare to a spherical one are reported. (PDF) [file pcbi.1004339.s001.pdf]

S1 Text

Advantage of a spherical eye

The optic-flow field is a two-dimensional vector field, where each vector is the apparent velocity of the images of objects on the eye of the agent. The optic-flow field experienced during translation results from the product of the relative nearness of objects in the environment and a factor depending on the angle between the direction of self-motion and the viewing direction of these objects ("viewing angle"):

$$\mathbf{OF} = -v\mu \left(\mathbf{u} - A \begin{pmatrix} \cos\epsilon\cos\phi \\ \cos\epsilon\sin\phi \\ \sin\epsilon \end{pmatrix} \right) \tag{1}$$
$$A = u_{\hat{x}}\cos\epsilon\cos\phi + u_{\hat{y}}\cos\epsilon\sin\phi + u_{\hat{z}}\sin\epsilon$$

where, $\epsilon \in [-\frac{\pi}{2}, +\frac{\pi}{2}]$, and $\phi \in [0, 2\pi]$ are the elevation and the azimuth of the agent's eye, respectively, μ is the nearness between the object and the agent in the direction (ϵ, ϕ), v is the speed of the agent and $\mathbf{u}$ is the direction of motion. The direction of motion is usually expressed as an earth coordinate system, i.e. Cartesian coordinates. However, an agent measures the optic flow in its eye referential system. We assume that the eye is spherical. The optic-flow field, therefore, needs to be expressed in spherical coordinates. A vector is conserved by a change of the referential system; however, its components change according to the elementary vectors' transformation. In the case of the transformation from Cartesian to spherical coordinates, the conservation of the vectors is expressed as:

$$\mathbf{OF} = OF_{\hat{x}}\hat{x} + OF_{\hat{y}}\hat{y} + OF_{\hat{z}}\hat{z} = OF_{\hat{\rho}}\hat{\rho} + OF_{\hat{\epsilon}}\hat{\epsilon} + OF_{\hat{\phi}}\hat{\phi} \tag{2}$$

where, $\mathbf{OF}$ is a vector, OF_x, OF_y, OF_z the component along $\hat{x}, \hat{y}, \hat{z}$, respectively, and $OF_\rho, OF_\epsilon, OF_\phi$ the component along $\hat{\rho}, \hat{\epsilon}, \hat{\phi}$, respectively.

The transformation from Cartesian coordinates to spherical coordinates is:

$$\begin{pmatrix} \hat{\rho} \\ \hat{\epsilon} \\ \hat{\phi} \end{pmatrix} = \begin{pmatrix} \cos\epsilon\cos\phi & -\sin\epsilon\cos\phi & -\sin\phi \\ \cos\epsilon\sin\phi & -\sin\epsilon\sin\phi & +\cos\phi \\ \sin\epsilon & +\cos\epsilon & 0 \end{pmatrix}^T \begin{pmatrix} \hat{x} \\ \hat{y} \\ \hat{z} \end{pmatrix} \tag{3}$$

Thus, the components in spherical coordinates are a function of the ones in the Cartesian coordinates:

$$\begin{cases} OF_{\hat{\rho}} & = +OF_{\hat{x}}\cos\epsilon\cos\phi & +OF_{\hat{y}}\cos\epsilon\sin\phi & +OF_{\hat{z}}\sin\epsilon \\ OF_{\hat{\epsilon}} & = -OF_{\hat{x}}\sin\epsilon\cos\phi & -OF_{\hat{y}}\sin\epsilon\sin\phi & +OF_{\hat{z}}\cos\epsilon \\ OF_{\hat{\phi}} & = -OF_{\hat{x}}\sin\phi & +OF_{\hat{y}}\cos\phi & \end{cases} \tag{4}$$

Applying the transformation above to optic-flow field vectors, the component in the spherical coordinates are:

$$\begin{cases} OF_{\hat{\rho}} & = 0 \\ OF_{\hat{\epsilon}} & = \frac{v}{p}\left(u_{\hat{x}}\sin\epsilon\cos\phi + u_{\hat{y}}\sin\epsilon\sin\phi - u_{\hat{z}}\cos\epsilon\right) \\ OF_{\hat{\phi}} & = \frac{v}{p}(u_{\hat{x}}\sin\phi - u_{\hat{y}}\cos\phi) \end{cases} \tag{5}$$

The radial component is obviously null, because the optic flow is the apparent motion on the eye. The components along the elevation and azimuth contain information about the relative nearness, but this information is entangled with that on self-motion. We want to extract the relative nearness from this system of equations without knowing the direction of motion $(u_x, u_y, u_z)^T$. Let us assume, however, that the direction of motion

is constrained to the equatorial plane, i.e. $u_z = 0$. By elevating the system of equations to the square and applying trigonometric identities, it can be shown for $\epsilon \neq 0$ that:

$$(v\mu)^2 = \frac{\sin(\epsilon)^2 OF_{\hat{\phi}}^2 + OF_{\hat{\epsilon}}^2}{\sin(\epsilon)^2} \tag{6}$$

We have used, until now, only the sum of both lines in the system of equations. By expressing $\mathbf{u}$ in polar coordinates, i.e. $\mathbf{u} = (\cos\theta, \sin\theta, 0)$, and using the ratio of $OF_\epsilon(u_z = 0)$ and $OF_\phi(u_z = 0)$, we get a factor telling how far away from the FOE and FOC we are looking, i.e. $\cos(2(\theta - \phi))$

$$\frac{1 - \cos(2(\theta-\phi))}{OF_{\hat{\phi}}^2} = \sin(\epsilon)^2 \frac{1 + \cos(2(\theta-\phi))}{OF_{\hat{\epsilon}}^2}$$
$$\Rightarrow \begin{cases} \cos(2(\theta-\phi)) & = \dfrac{1 - h(\epsilon)}{1 + h(\epsilon)} \\ h(\epsilon) & = \sin(\epsilon)^2 \dfrac{OF_{\hat{\phi}}^2}{OF_{\hat{\epsilon}}^2} \end{cases} \tag{7}$$

The term $\cos(2(\theta - \phi))$ is independent of ϵ. It is, therefore, a constant for a given ϕ and θ. Thus, this factor can be computed for $\widetilde{\epsilon} \neq 0$ and used in the following equation to compute $v\mu$ for $\epsilon = 0$:

$$(v\mu)^2 = OF_{\hat{\phi}}^2 \frac{1 + h(\widetilde{\epsilon})}{h(\widetilde{\epsilon})} \tag{8}$$

This equation holds as long as the optic flow is not zero and the term $\cos(2(\theta - \phi)$ can be computed, i.e an $\epsilon \neq 0$ exists for a given ϕ.

Finally, the relative nearness can be computed from the optic-flow field experienced during a translation in the null elevation plane with the following equation, $\forall \epsilon, \widetilde{\epsilon} \neq 0$:

$$\boxed{(v\mu(\phi,\epsilon))^2 = OF(\phi,\epsilon)_{\hat{\phi}}^2 \left(1 + \frac{OF(\phi,\widetilde{\epsilon})_{\hat{\epsilon}}^2}{OF(\phi,\widetilde{\epsilon})_{\hat{\phi}}^2} \frac{1}{\sin(\widetilde{\epsilon})^2}\right)} \tag{9}$$

Error on relative nearness

The motion of the agent has been assumed to be contained in the null elevation plane of the agent. The agent might not exactly move in this plane and, therefore, the upward component of the motion u_z will be different from zero. If the agent estimates the relative nearness with our set of equations, derived with $u_z = 0$, it will make an error proportional to u_z, but this error also depends on the viewing direction (ϵ, ϕ) and the forward and sideward component of the motion (u_x, u_y)

$$\frac{\sin(\epsilon)^2 OF_{\hat{\phi}}^2 + OF_{\hat{\epsilon}}^2}{\sin(\epsilon)^2} = (v\mu)^2 \left(1 - 2u_{\hat{z}}\text{cotan}\epsilon(u_{\hat{x}}\cos\phi + u_{\hat{y}}\sin\phi) + u_{\hat{z}}^2(\text{cotan}^2\epsilon - 1)\right) \tag{10}$$

Note: $u_z \in [-1, 1]$, because $\|\mathbf{u}\| = 1$, therefore, $u_z^2 < u_z$.

Limitation of a cylindrical eye

The relative nearness can be extracted by equation (#Eq: 12#), as long as the agent translates in the null elevation plane and has a spherical eye. However, for an agent with a cylindrical eye, the components of the optic flow in cylindrical coordinates are:

$$\begin{cases} OF_{\hat{\rho}} & = 0 \\ OF_{\hat{z}} & = -\frac{v}{p} u_{\hat{z}} \\ OF_{\hat{\phi}} & = +\frac{v}{p}(u_{\hat{x}}\sin\phi - u_{\hat{y}}\cos\phi) \end{cases} \tag{11}$$

where, ρ,z,ϕ are the cylindrical components, $\vec{u} = (u_x, u_y, u_z)^T$ and v the direction of motion and the speed of the agent, respectively, and p the distance to the closest object in the viewing direction defined by ρ,z,ϕ. The motion of the agent with a spherical eye has been constrained in the null elevation plane. This constraint for a cylindrical eye is a motion in a plane parallel to the base of the cylinder, i.e. $u_z = 0$. However, applying this constraint to the previous equation leads to $OF_z = 0$. The system has one equation and three unknown variables, therefore, it cannot be solved. Constraining the motion in a plane parallel to the base of the cylinder is, thus, not a suitable strategy to extract nearness from optic flow measured on a cylindrical eye.

References
