# Supplementary material for: A Bio-inspired Collision Avoidance Model Based on Spatial Information Derived from Motion Detectors Leads to Common Routes
Source: PLoS Comput Biol. 2015 Nov 19;11(11):e1004339. doi: 10.1371/journal.pcbi.1004339 (PMC4652890; doi:10.1371/journal.pcbi.1004339)
Supplement: S2 Text — The performance is quantified in term of its reliability, i.e. the percentage of trajectories with a collision occurring before the goal is reached, and its efficiency, i.e. the distance traveled needed to reach the goal. The later is compared to the shortest path, and a collision proof path, both derived from the object positions in the environment. (PDF) [file pcbi.1004339.s002.pdf]

S2 Text

Performance of the algorithm in cluttered environments

The performance of our collision avoidance algorithm combined with a goal direction was assessed and quantified in term of its reliability, i.e. the percentage of trajectories with a collision occurring before the goal is reached, and its efficiency, i.e. the distance traveled needed to reach the goal. The algorithm was tested in two cluttered environments, either with or without ceiling and outer walls, and either covered with a 1 mm or 4 mm random checkerboard pattern. For each cluttered environment, 201 starting positions were tested. For the overall 1,608 simulated trajectories, only three crashes were observed. Thus, our collision avoidance algorithm is reliable under the conditions tested.

The distance traveled is a poor measurement to quantify the efficiency of the algorithm, because it depends on the layout and dimension of the environment, the distance separating the starting and goal location, as well as the number of obstacles between those locations. Therefore, the efficiency of the algorithm was quantified as the ratio between the theoretically shortest trajectory between the starting and goal location and the distance traveled. The problem of finding the theoretically shortest trajectory is linked to graph theory, and is solved in this context by Dijkstra's algorithm [1]. Therefore, the environment was transferred to a graph-like representation, here the goal and starting location, and the corners of the obstacles were the nodes of the graph. Two nodes in the graph were connected if the line between those nodes was not crossing an object. The shortest path was computed using Dijkstra's algorithm [1]. This method is similar to allowing an agent to get as close as possible to an object. Our collision avoidance algorithm, however, prevented such behavior. Therefore, a second reference had to be calculated. A Voronoi diagram [2, p. 513-529] is composed of segments maximizing the distance between two neighboring nodes. When the nodes represent the center of mass of the objects, the segments represent the safest trajectory between two neighboring objects. The environment can, therefore, be represented by a graph with the segments and the intersection between segments representing the edges and the nodes of the graph, respectively. However this graph needs to be connected to the starting and goal locations. These starting and goal locations were connected to the closest node in the graph, outside the obstacles. The shortest trajectory in the graph, which differs from the theoretically shortest trajectory in the environment, is called the "Voronoi trajectory." This trajectory has been computed using Dijkstra's algorithm [1]. Figure S2 shows the efficiency of our collision avoidance algorithm. Our algorithm is more efficient than the "Voronoi trajectories" in ~~most~~77.4% and 73.6% for the 4mm and 1mm pattern, respectively, of the tested starting conditions.

References

1. Dijkstra EW. A note on two problems in connexion with graphs. Numerische mathematik. 1959;1(1):269–271.

2. Goodman JE, O'Rourke J. Handbook of discrete and computational geometry. CRC press; 2010.
